# Segmentectomy vs Lobectomy for Peripheral Early-Stage Non-Small Cell Lung Cancer in Patients With Radiologically High Risk Tumors

**DOI:** 10.1016/j.atssr.2026.03.005

**Published:** 2026-03-24

**Authors:** Patrick Deniz Hurley, Nilanjan Chaudhuri, Joshil Lodhia, Richard Milton, Marco Nardini, Kostas Papagiannopoulos, Peter Tcherveniakov, Elaine Teh, Alessandro Brunelli

**Affiliations:** 1 Department of Thoracic Surgery, Leeds Teaching Hospitals NHS Trust, Leeds, United Kingdom; 2 University of Leeds, School of Medicine, Leeds, United Kingdom

## Abstract

**Background:**

The increasing use of segmentectomy for early-stage peripheral non-small cell lung cancer (NSCLC) raises questions about its oncologic adequacy compared with lobectomy, particularly for tumors with high-risk features. This study aimed to compare oncologic outcomes between segmentectomy and lobectomy for clinical stage IA1-2 peripheral NSCLC, stratified by radiologic tumor risk.

**Methods:**

This retrospective single-center study analyzed 527 patients with peripheral NSCLC ≤2 cm who underwent either lobectomy (n = 240), or segmentectomy (n = 287) from January 2017 to December 2024. High-risk tumors were defined as cT1b, solid dominant, and hypermetabolic (maximal standardized uptake value >2.5). Primary end points included 4-year overall survival (OS) and event-free survival (EFS). Cox regression analysis was performed to identify independent predictors of EFS.

**Results:**

High-risk tumors comprised 122 lobectomies (51%) and 73 segmentectomies (25%). For low-risk tumors, there was no significant difference in 4-year OS or EFS (OS: lobectomy 85% vs segmentectomy 84% [*P* = .74]; EFS: lobectomy 75% vs segmentectomy 72% [*P* = .74]). For high-risk tumors, 4-year OS and EFS were also not different after lobectomy or segmentectomy (OS: lobectomy 80% vs segmentectomy 68% [*P* = .18]; EFS: lobectomy 72% vs segmentectomy 60% [*P* = .33]). In the inverse probability weighting–adjusted Cox proportional hazards model, lobectomy was not independently associated with improved EFS (hazard ratio, 0.86; 95% CI, 0.59-1.25; *P* = .43).

**Conclusions:**

Segmentectomy demonstrates oncologically equivalent outcomes compared with lobectomy for both low-risk and high-risk clinical stage IA1-2 peripheral NSCLC. These findings support the oncologic safety of segmentectomy as an alternative to lobectomy in appropriately selected patients with small peripheral NSCLC, even in the high-risk tumor subset.

## Visual Abstract

**Figure:**
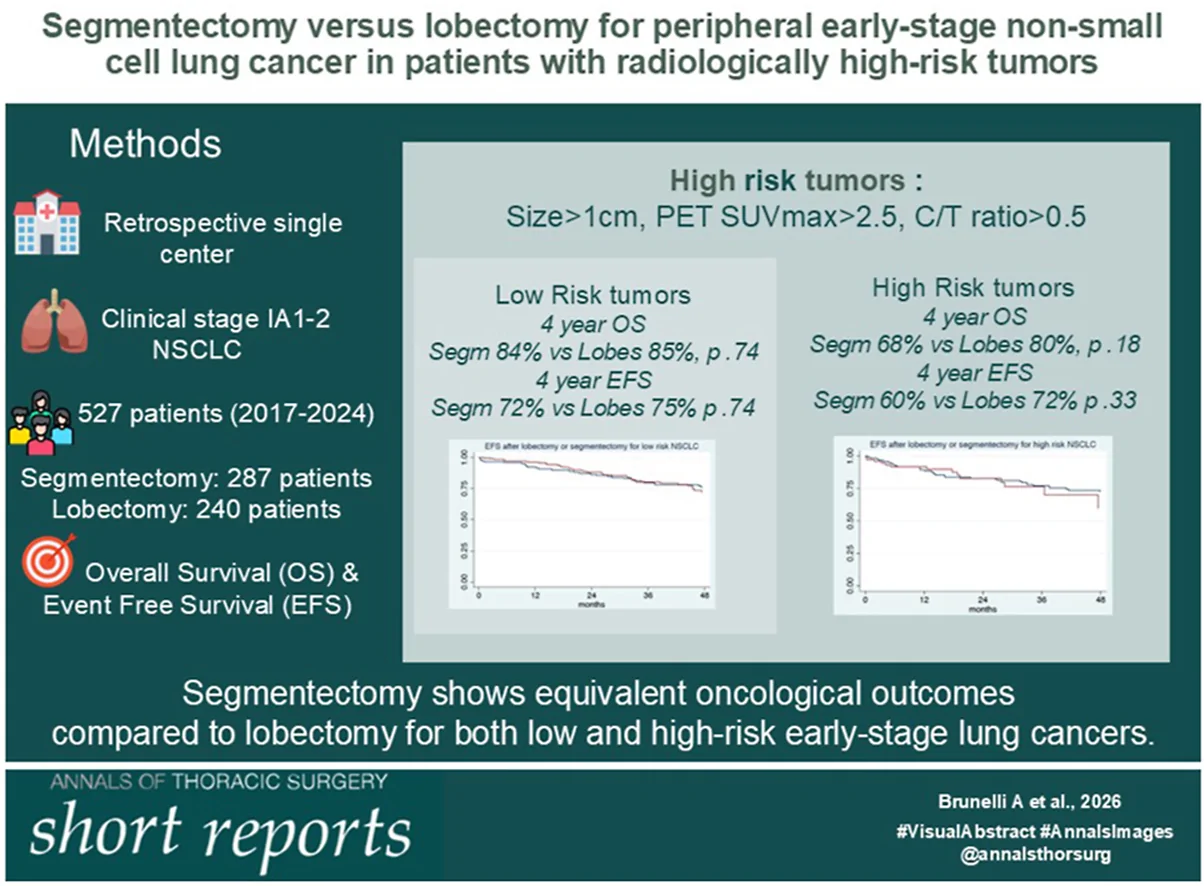

**In Short**

▪ The 4-year overall survival and event-free survival after segmentectomy for radiologically high risk tumors were like those after lobectomy.
▪ Inverse probability weighting–adjusted Cox model (propensity score including high-risk status) showed that the extent of resection was not associated with event-free survival.
▪ Segmentectomy demonstrates oncologically equivalent outcomes compared with lobectomy for high-risk clinical stage IA1-2 peripheral non-small cell lung cancer.

Segmentectomies are now generally considered noninferior to lobectomies for peripheral ≤2-cm non-small cell lung cancer (NSCLC) without known nodal disease. Some tumor radiologic features, such as size, consolidation to tumor ratio, and high positron emission tomography maximal standardized uptake value (SUVmax), among others, have been considered surrogates of a biologically more aggressive tumor and associated with poorer outcomes after lung cancer resections. Whether the oncologic equivalence of segmentectomy and lobectomy is valid in high-risk radiologic subgroups remains uncertain. In this study, we evaluated long-term oncologic outcomes of lobectomy and segmentectomy in clinical stage IA1-2 NSCLC, stratified by radiologic risk factors.

## Patients and Methods

### Study Design and Population

This single-center retrospective study was evaluated by the Leeds Teaching Hospitals Research and Innovation Department and determined to be service evaluation, not requiring formal ethics review; individual consent was waived. We reviewed a prospectively maintained database of patients with clinical stage IA1-2 NSCLC (cT1a-b N0 M0) treated between January 2017 and December 2024. Lobectomies included in this analysis were performed between 2017 and mid-2022, whereas segmentectomies continued through 2024, reflecting progressively wide institutional adoption of sublobar resection during the study period. Before 2022, more surgeons still favored lobectomy for stage I NSCLC in our unit, although few of them already performed segmentectomies in selected patients. After this date, the standard for a peripheral stage IA1-2 became a segmentectomy. Including lobectomies performed after this date would have introduced a greater selection bias.

Inclusion criteria were pathologically confirmed peripheral NSCLC, tumor size ≤2 cm, and negative preoperative nodal evaluation by positron emission tomography/computed tomography and endobronchial ultrasound where indicated. All cases were reviewed in a multidisciplinary tumor board. Preoperative assessment included pulmonary function testing and fitness assessment according to established guidelines.

Eight thoracic surgeons performed all procedures. Segmentectomy was defined as anatomic resection involving division of at least segmental arteries and bronchus, with stapled division of intersegmental planes. All patients underwent systematic lymph node dissection.

No frozen sections on lymph nodes or margins were performed in this series per institutional policy.

### Outcomes and Definitions

Patients were observed through March 2025. No patient was lost to follow-up. Primary outcomes included 4-year overall survival (OS), event-free survival (EFS), lung cancer–specific survival (LCSS), and recurrence patterns. OS was defined as time from surgery to death from any cause; EFS, as time to recurrence or death; and LCSS, as time to death from lung cancer.

Locoregional recurrence was defined as recurrence within the ipsilateral lung, hilar nodes, or mediastinum; all other recurrences were systemic. Cardiopulmonary complications included pneumonia, atelectasis requiring bronchoscopy, pulmonary edema, acute lung injury, respiratory insufficiency requiring ventilation, atrial fibrillation, myocardial ischemia, pulmonary embolism, and cerebrovascular events.

A high-risk tumor was defined as fulfilling all 3 radiologic criteria: cT1b (>1 cm), solid-dominant morphology (consolidation to tumor ratio >0.5), and SUVmax >2.5. The radiologic criteria were selected according to previous investigations.^1^ For the purpose of this study, we omitted pathologic high-risk criteria as we wanted to represent a scenario in which the practicing surgeon should decide the extent of resection preoperatively. The SUVmax threshold was based on prior institutional analysis identifying 2.5 as the optimal predictor of locoregional recurrence.^2^ Tumors lacking 1 or more features were classified as low risk.

The statistical analysis is reported in the Supplemental Material.

## Results

We included 527 patients with clinical stage IA1-2 NSCLC (240 lobectomies and 287 segmentectomies). The Table shows the characteristics of the patients in the study. Patients undergoing lobectomy more frequently had cT1b, hypermetabolic, and more solid tumors compared with those undergoing segmentectomy. Baseline characteristics were substantially similar.

**Table:** Characteristics of Patients Included for the Analysis

Variable	Lobectomies (n = 240)	Segmentectomies (n = 287)	*P* Value
Age, y	69.1 (7.8)	69.7 (7.9)	.43
Sex, male	106 (44%)	103 (36%)	.053
BMI, kg/m^2^	26.9 (5.2)	27.1 (5.0)	.35
FEV_1_, %	89.4 (20.5)	92.2 (21.0)	.17
Dlco, %	74.9 (17.4)	79.5 (18.6)	.001
CCI >2	31 (13)	30 (10)	.38
cT1b	178 (74)	176 (61)	.002
PET SUVmax >2.5	180 (75)	157 (55)	<.001
C/T ratio >0.5	180 (75)	157 (55)	<.001
Adenocarcinoma	184 (77)	228 (79)	.44

Results are expressed as average (SD) for numeric variables or count and percentages for categorical ones. BMI, body mass index; CCI, Charlson comorbidity index; C/T ratio, consolidation to tumor ratio; Dlco, carbon monoxide lung diffusion capacity; FEV_1_, forced expiratory volume in 1 second; PET, positron emission tomography; SUVmax, maximal standardized uptake value.

Most of the procedures were performed minimally invasively (95% of lobectomies and 99% of segmentectomies). Conversion rate was 7% in lobectomies and 3.2% in segmentectomies (*P* = .19). The incidence of cardiopulmonary morbidity was 18% after lobectomies and 17% after segmentectomies (*P* = .98). Ninety-day mortality rates were 3.3% (8/240 patients) after lobectomy and 1.4% (4/287 patients) after segmentectomy (*P* = .15). Median postoperative hospital stay was 1 day shorter after segmentectomy (4 days vs 5 days; *P* = .0002).

The results of the analysis of radiologic risk factors in the entire population are reported in the Supplemental Material.

Median follow-up was 33.3 months (interquartile range [IQR], 17-53 months) in the segmentectomy patients compared with 80 months (IQR, 60-89 months) in the lobectomy patients.

### Comparison of Lobectomy and Segmentectomy in Low-Risk Tumors

**Figure 1:**
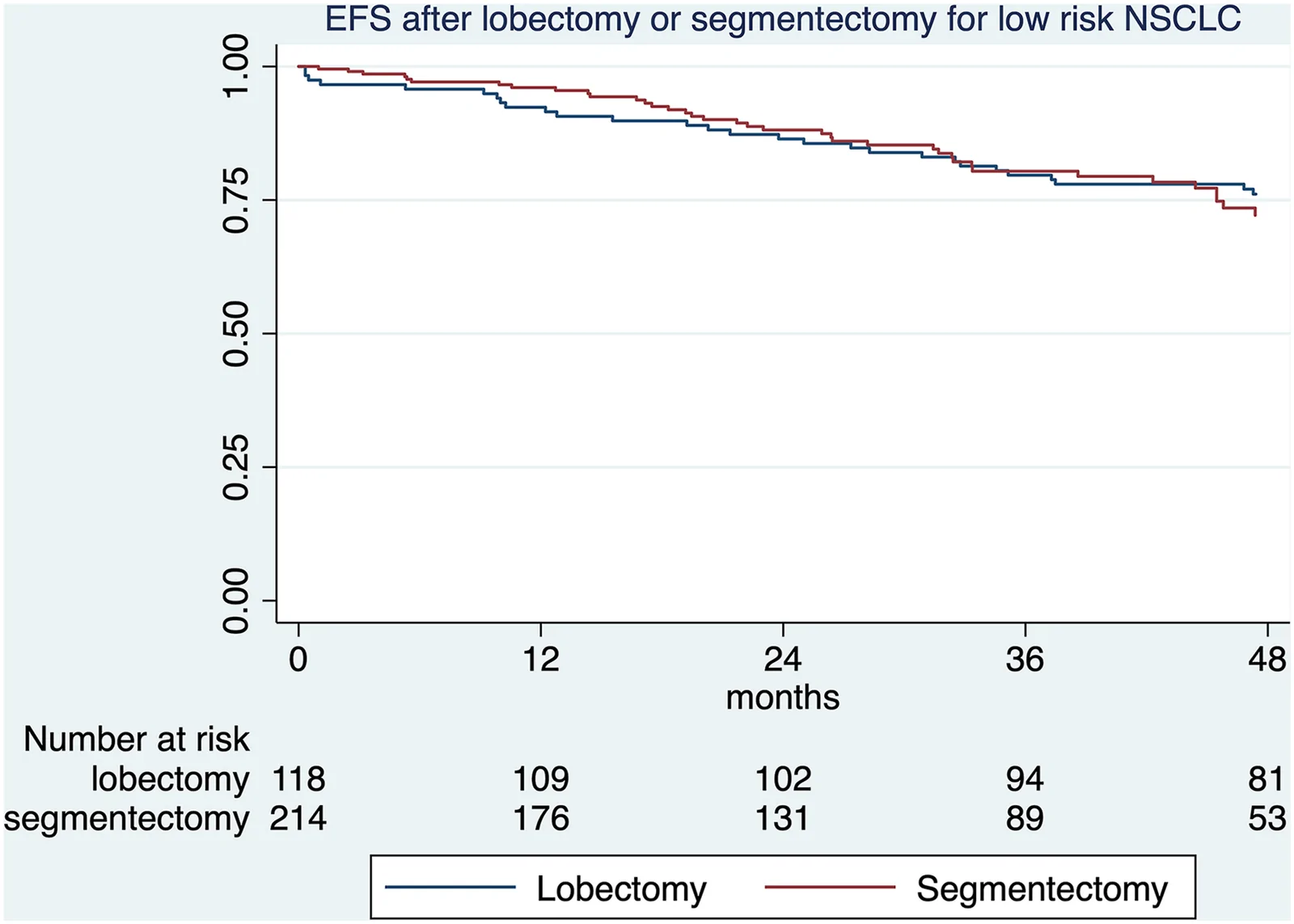
Kaplan-Meier survival estimates of 4-year event-free survival (EFS) after lobectomy or segmentectomy in patients with low-risk tumors. (NSCLC, non-small cell lung cancer.)

According to our predefined criteria, there were 118 lobectomies and 214 segmentectomies in this category. The 4-year OS was similar after lobectomy and segmentectomy: 85% (95% CI, 77-90) vs 84% (95% CI, 75-89; *P* = .74). The 4-year EFS was also not different: 75% vs 72% (*P* = .74; Figure 1).

The 4-year LCSS was 94% (95% CI, 87-97) after lobectomy and 96% (95% CI ,89-98) after segmentectomy (*P* = .073). Median resection margins after lobectomy and segmentectomy were 30 mm (IQR, 15-42 mm) and 10.5 mm (IQR, 5-20 mm), respectively (*P* < .001). Despite a higher incidence of compromised margins after segmentectomy (11% vs 4.2%; *P* = .041), locoregional recurrence was similar (15% vs 10%; *P* = .22). Nodal upstaging was also similar (4.2% vs 3.7%; *P* = .78).

### Comparison of Lobectomy and Segmentectomy in High-Risk Tumors

**Figure 2:**
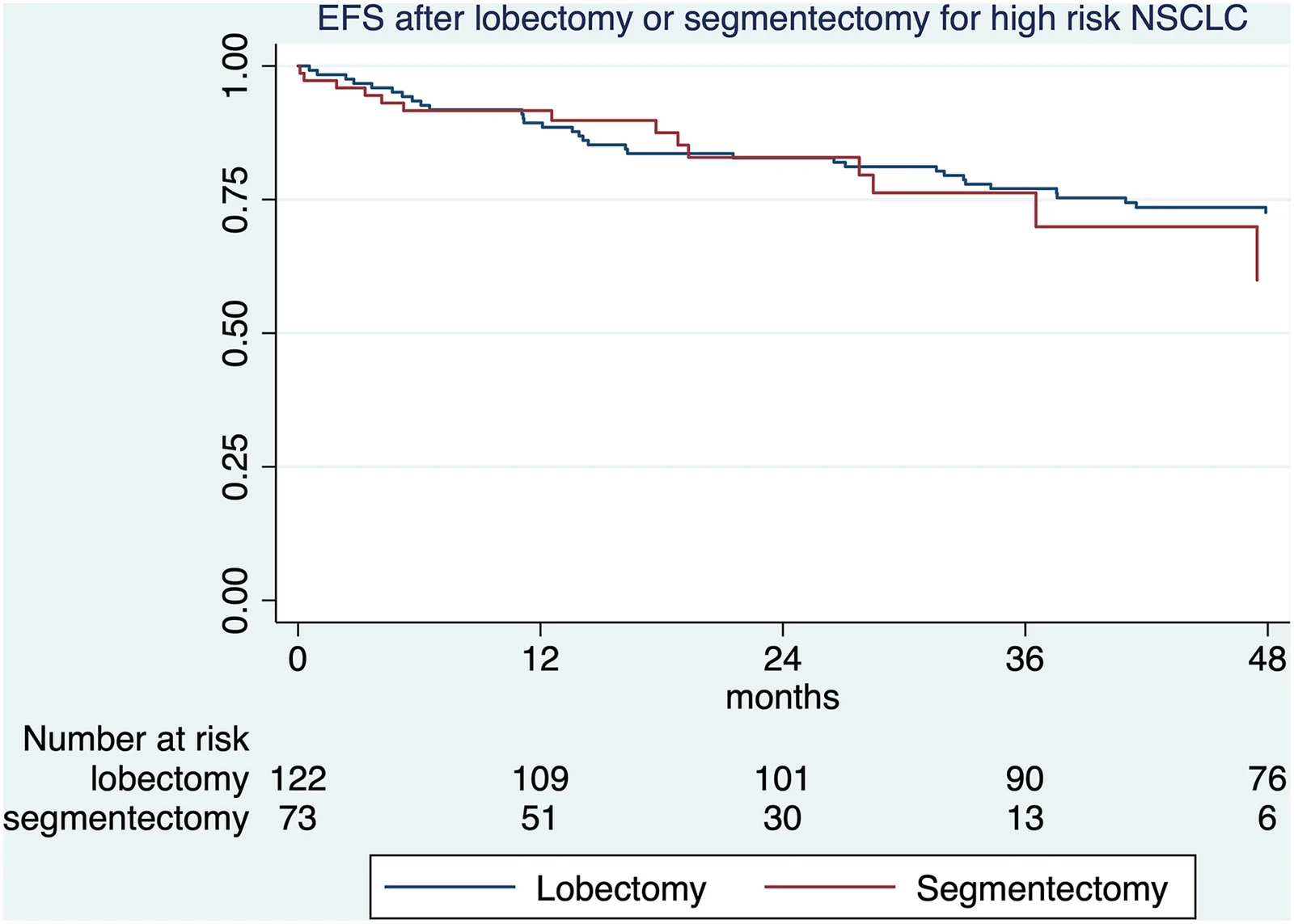
Kaplan-Meier survival estimates of 4-year event-free survival (EFS) after lobectomy or segmentectomy in patients with high-risk tumors. (NSCLC, non-small cell lung cancer.)

There were 122 lobectomies and 73 segmentectomies in this category. We found no difference in 4-year OS between lobectomy (80%; 95% CI, 72-87) and segmentectomy (68%; 95% CI, 42-84; *P* = .18.) The 4-year EFS was similarly not different: 72% vs 60% (*P* = .33; Figure 2).

**Figure 3:**
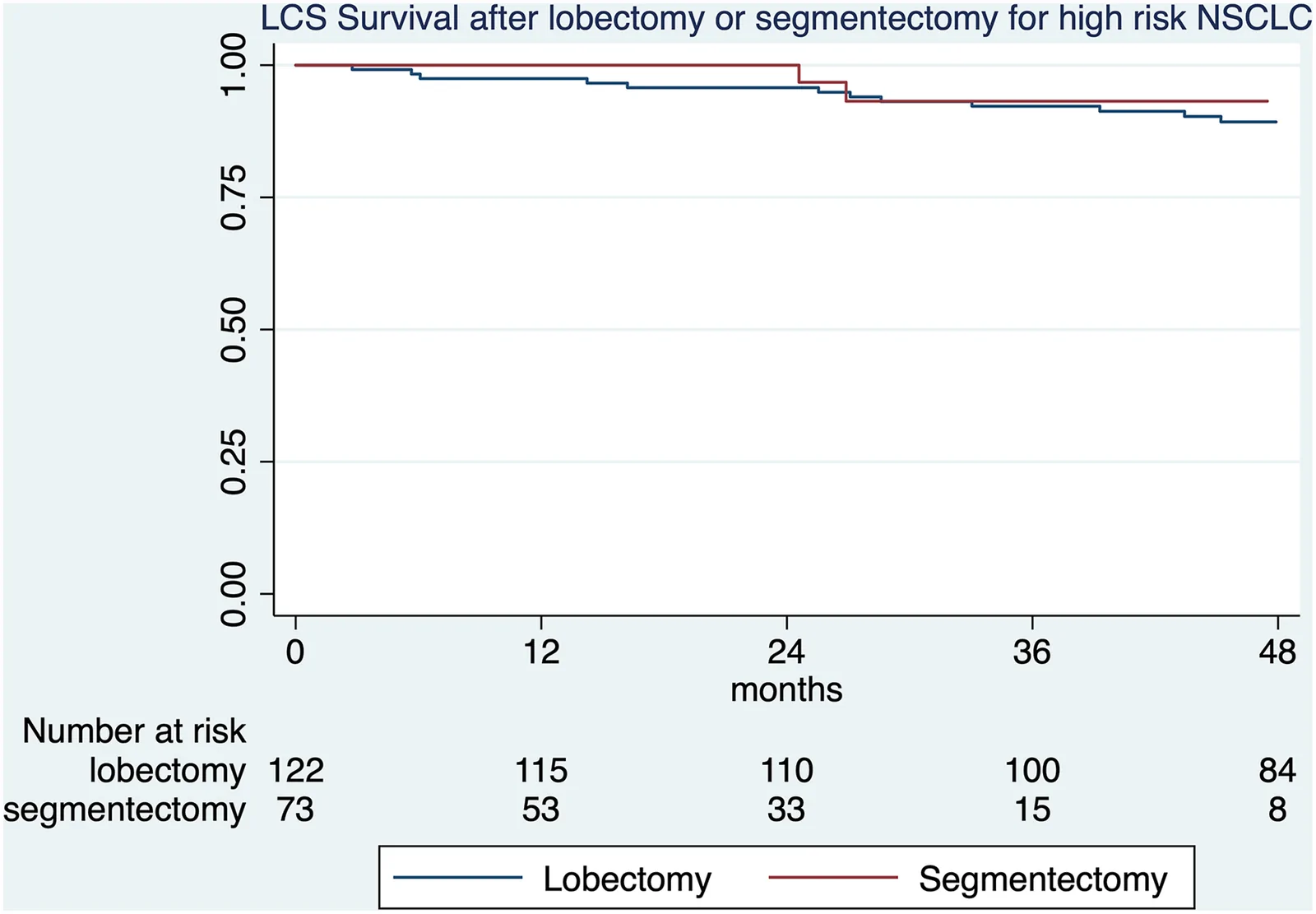
Kaplan-Meier survival estimates of 4-year lung cancer–specific (LCS) survival after lobectomy or segmentectomy in patients with high-risk tumors. (NSCLC, non-small cell lung cancer.)

Four-year LCSS was 88% (95% CI, 81-93) after lobectomy and 93% (95% CI, 75-98) after segmentectomy (*P* = .37; Figure 3). Median resection margins after lobectomy and segmentectomy were 28.5 mm (IQR, 15.5-40 mm) and 12 mm (IQR, 0.5-20 mm), respectively (*P* < .001). The rate of compromised margins was similar (9.6% after segmentectomy and 11% after lobectomy; *P* = .01). Locoregional recurrence developed in 13% after lobectomy and 5.4% after segmentectomy (*P* = .14). Nodal upstaging occurred in 16% vs 9.6% (*P* = .21). Compromised margins were similar (11% vs 9.6%; *P* = 1).

### Regression Analyses

A Cox proportional hazards model weighted by inverse probability of treatment weighting and fitted using a propensity score that included high-risk status of the covariates showed that lobectomy was not independently associated with improved EFS (hazard ratio, 0.86; 95% CI, 0.59-1.25; *P* = .43). In Fine-Gray competing risk analysis, lobectomy was the only independent predictor of lung cancer–specific death (subdistribution hazard ratio, 2.54; *P* = .021; 95% CI, 1.1-5.6).

## Comment

In this single-center study of clinical stage IA1-2 peripheral NSCLC, anatomic segmentectomy demonstrated oncologic outcomes equivalent to lobectomy across both low-risk and high-risk radiologic subgroups. Four-year OS, EFS, and LCSS did not differ significantly between procedures, suggesting that segmentectomy is an oncologically safe alternative to lobectomy when it is performed with appropriate patient selection and oncologic technique.

Our findings differ from those of Caso and coworkers,^1^ who reported that patients with 2 or more radiologic high-risk features experienced lower recurrence risk after lobectomy compared with sublobar resection. A key distinction is the inclusion of nonanatomic wedge resections (representing 55% of sublobar procedures in their analysis), whereas our study examined only anatomic segmentectomy. Previous studies have demonstrated that segmentectomy is associated with better nodal harvesting, larger resection margins, and improved recurrence-free survival compared with wedge resection.^3^^,^^4^ Despite that the debate is still open on the relative merits of wedge vs segmentectomies,^5^ we prefer to keep segmentectomy distinct from wedge resection when interpreting comparative data.

We observed a higher rate of compromised margins (pRx/R1) after segmentectomy in the low-risk group, although this did not result in higher locoregional recurrence. Margin status remains an important predictor of recurrence; but when adequate margins are achieved, segmentectomy appears equivalent to lobectomy in oncologic performance.^6^ Our findings support the feasibility of obtaining oncologically sound margins even in segmentectomy for small peripheral tumors.

We selected radiologic criteria including cT1b size, solid morphology, and SUVmax >2.5 because of their established associations with tumor aggressiveness and prognosis. Solid-dominant lesions on high-resolution computed tomography correlate strongly with invasive adenocarcinoma and worse outcomes.^7^ Elevated SUVmax is associated with higher grade histologic type and increased recurrence risk.^8^ Nakamura and coworkers^9^ demonstrated that SUVmax also predicts histologic subtype, with micropapillary-predominant adenocarcinoma showing the highest uptake—subtypes known to portend poorer prognosis. Because preoperative histologic subtyping is often limited by sampling error, radiologic features provide practical guidance for preoperative risk stratification. Radiologic features have shown a promising association with pathologic aggressiveness of the tumor. Interestingly, 2 of the most consistent factors associated with spread through air spaces are a solid morphology and a larger size, with a consolidation to tumor ratio >0.5 reported to be associated with spread through air spaces in 82% of cases.^10^ These 2 factors are incorporated in our definition of high risk.

Interestingly, we found a 2-fold higher risk of lung cancer–specific death after lobectomy. This finding may be explained in part by the presence of unrecognized confounders and selection bias inherent with the variable “lobectomy,” which serves as a surrogate for a more aggressive tumor. Alternatively, a lobectomy may be associated with a lower chance to receive radical treatment in case of relapse or second lung cancer because of a more profound physiologic effect compared with segmentectomy.

This study has limitations. As a retrospective single-center analysis, external validity is limited, and selection bias is possible, particularly as high-risk tumors were more frequently treated with lobectomy. Although multivariable and competing risk analyses were used to adjust for imbalances, unmeasured confounders may remain. In particular, the subjective surgeon’s judgment to decide one procedure over the other is difficult to record in a retrospective analysis. Follow-up duration was shorter for segmentectomy patients, reflecting evolving practice patterns. Finally, because of the limited number of events, the study had low power for EFS comparisons, which may have increased the risk of a type II error. In this regard, the analysis may have been underpowered to detect modest but clinically meaningful differences between groups. The absence of a statistical significance should be interpreted with caution owing to small sample size and unbalanced follow-up. These findings need confirmation in larger samples and with longer follow-up durations.

Finally, radiologic risk factors should be integrated with biologic and pathologic risk factors (visceral pleura invasion, large cell neuroendocrine variants, lymphovascular invasion, tumor differentiation, genomic mutations) to generate a more precise risk stratification that could guide the selection of the most appropriate extent of resection.

Considering the several limitations and selection bias of this analysis, we found that anatomic segmentectomy did not show poorer oncologic outcomes compared with lobectomy for peripheral clinical stage IA NSCLC, including for tumors with high-risk radiologic features. When performed with careful patient selection and adequate margins, segmentectomy offers a safe and effective parenchyma-sparing alternative to lobectomy in modern thoracic surgery.
