# Supplementary material for: Segmentectomy vs Lobectomy for Peripheral Early-Stage Non-Small Cell Lung Cancer in Patients With Radiologically High Risk Tumors
Source: Ann Thorac Surg Short Rep. 2026 Mar 24;4(3):932–7. doi: 10.1016/j.atssr.2026.03.005 (PMC13555413; doi:10.1016/j.atssr.2026.03.005)
Supplement: Supplementary file 1 [file mmc1.docx]

***Supplement***

***Statistical Analysis***

Demographic, radiological, and pathological data were collected from electronic medical records. Tumour CTR was measured on CT and classified as pure ground-glass opacity (C/T = 0), part-solid (0 < C/T < 1), or pure solid (C/T = 1). SUVmax was derived from PET imaging.

Continuous variables were compared using Student’s t-test or Mann–Whitney U test; categorical variables using Chi-square or Fisher’s exact test. Kaplan–Meier analysis was used for survival outcomes, truncated at 4 years.

To reduce confounding due to non-random treatment allocation (lobectomy vs segmentectomy), inverse probability of treatment weighting (IPTW) based on the propensity score was applied. The propensity score for undergoing a lobectomy (as opposed to segmentectomy) was estimated using a logistic regression model including the following variables: radiologic risk group, age, sex, FEV₁, DLCO, BMI, histology, and Charlson’s Comorbidity Index. Weighted Cox proportional hazards regression with robust variance estimators were fitted to evaluate the association between the type of resection and event-free survival (EFS), and the hazard ratio and 95% confidence intervals were reported.

Lung cancer–specific mortality was analysed using a Fine–Gray competing-risk model adjusting for the same risk factors listed above. Analyses were performed using Stata 15.1 (StataCorp, TX).

**Radiological Risk Factor Analysis**

Fifty-one percent (122 of 240) of tumours in the lobectomy group showed radiological high-risk features, compared to 25% (73 of 287) in the segmentectomy group (p<0.0001).

Tumours in the lobectomy group showed 1 high-risk factor in 18% of patients, 2 high-risk factors in 27% and all 3 high-risk factors in 51% of patients. Ninety-five percent of patients had at least one high-risk factor.

Tumours in the segmentectomy group displayed 1 radiological high-risk factor in 26% of patients, 2 high-risk factors in 34% and all 3 high-risk factors in 25% of patients. Eighty-six percent of patients had at least one high-risk factor.

To check for redundancy of high-risk factors, we analysed their relative distributions. There were 337 solid dominant tumours, 75% of them were hypermetabolic. In addition, there were 354 cT1b tumours, 72% of them were hypermetabolic.

The 4-year OS was 88% (95% CI 79-93) in patients with one high-risk factor, 81% (95% CI 73-86) in those with two risk factors and 79% (95% CI 71-85) in patients with three risk factors (p=0.27). The 4-year OS of patients without any high-risk factor (94%, 95% CI 75-99) was better than in those with any number of risk factors (82%, 95% CI 77-85, p<0.001).

In the entire population the 4-year EFS was similar in patients with one, two or three high-risk factors: 74% (95% CI 63-82) with one risk factor, 71% (95% CI 62-78) with two risk factors and 70% (95 CI 62-77) with all three risk factors (p=0.65). The 4-year EFS was 89% (95% CI 71-96) in patients with tumours displaying no high-risk factors compared to 72% (95% CI 67-76) in those with tumours displaying any high-risk factor (p=0.029).

To rule out the presence of unrecognized changes in the management system which may have affected the outcome independent of the adoption of segmentectomy, we performed a sensitivity analysis to check whether there was any difference in occurrences of events (any death or recurrence) between an early period (2017-2019) and a more recent one (2020-2023) allowing for a balanced follow up (events occurred within two year from surgery). We could not detect any differences in events rate (13% vs. 15%, p=0.55).
